# Beyond the Birds and Bees: Why Comprehensive Sexuality Education Is a Game-Changer for Everyone

**DOI:** 10.7759/cureus.89479

**Published:** 2025-08-06

**Authors:** Arwinder Singh, Amit Jangid, Devender P Yadav, Yashpal S, Salu Chandran, Anand Kumar, Ramit Sai

**Affiliations:** 1 Forensic Medicine and Toxicology, All India Institute of Medical Sciences, Rishikesh, Dehradun, IND; 2 Forensic Medicine and Toxicology, Maulana Azad Medical College, New Delhi, IND

**Keywords:** comprehensive sexuality education, consent, disabilities, lgbtq+, public health, relationships, sexual rights, teacher training

## Abstract

Comprehensive sexuality education (CSE) is a vital public health intervention that extends beyond basic biological facts to encompass emotional well-being, healthy relationships, and consent. This review explores the evolving landscape of sexuality education, examining diverse teaching methods, cultural and societal influences, and evidence-based strategies for effective implementation across all age groups and settings. By highlighting innovative practices, addressing persistent gaps and controversies, and advocating for inclusive models, this article positions sexuality education as a critical component of holistic health and public well-being, essential for fostering informed decision-making and promoting sexual rights globally.

## Introduction and background

Sex education is often limited to biology and preventing unwanted pregnancies or sexually transmitted infections (STIs). But there is more, where youth learns to build healthy relationships, understand their emotions, advocate for themselves, and develop a positive body image. This holistic approach is comprehensive sexuality education (CSE) [1].

CSE is an educational process structured to impart knowledge, skills, attitudes, and values in the cognitive, emotional, physical, and social dimensions of sexuality [1,2]. Its objective is to empower children and young people to achieve optimal health (physical as well as psychological), well-being, and dignity. This includes developing respectful social and sexual relationships, understanding the impact of their choices, and asserting their sexual rights in their lives. CSE emphasizes on positive view of sexuality with fundamental values including respect, inclusion, non-discrimination, equality, empathy, responsibility, and reciprocity. It also promotes constructive attitudes toward bodies, puberty, relationships, sex, and family life by moving beyond a narrow, "just the facts" approach to sexuality and framing sexuality as a natural part of the human body [1].

CSE integrates accurate details on human development, anatomy, reproductive health, contraception, childbirth, and STIs (including HIV). It also addresses the associated psychological, social, and emotional complexities [1].

The incremental pedagogical approach of CSE establishes foundational knowledge and skills from an early age through a spiral curriculum [1]. The spiral curriculum ensures the annual revision of topics with new information, building upon prior learning. This approach ensures age and developmentally appropriate content, which is comprehensive and extends beyond just sexual behaviors. This broadens the definition of CSE, making it a public health intervention that supports overall human development and societal flourishing [3]. CSE represents a significant shift from a narrow disease prevention model to a more expansive public health framework. This implies that sexual health is not simply the absence of STIs or unintended pregnancies but rather a holistic state of physical, mental, and social well-being related to sexuality.

## Review

This is a scoping review of the literature on CSE. Our search strategy included a broad sweep of academic databases, including PubMed and Google Scholar, to identify key studies, meta-analyses, and policy documents published in the last decade. The literature was selected based on its relevance to the core components of CSE and its reported outcomes.

The evolution of sexuality education

The conventional sexuality education initiatives were "health-based" programs aimed at preventing sexual risks and adverse outcomes (STIs, HIV infections, and unintended pregnancies). Early programs provided information on reproductive physiology along with various contraceptive and protective methods. Conventional CSE advocated for all available strategies to mitigate sexual risks, not just the abstinence-only approach. Behavior change theory was the theoretical foundation for these programs [3].

However, a narrow focus on risk and health outcomes proved to be insufficient to address the intricate realities related to sex and relation including the developmental needs of young people. This led to a significant paradigm shift toward an empowerment-directed and rights-based approach to CSE. This contemporary approach adopts a positive view of sexuality, acknowledging young people as sexual beings with inherent feelings and desires [3]. Notably, the Convention on the Rights of the Child (1990) affirmed sexuality education itself as a fundamental human right [4].

A major development in this evolution is the integration of gender and rights as core components of CSE. This aims to cultivate non-sexist attitudes, promote gender equality, and foster safe, consensual, and egalitarian relationships [3]. Key concepts within rights-based CSE curricula include relationships, values, rights, culture, gender understanding, violence prevention, health skills, human body and development, sexuality and sexual behavior, and sexual and reproductive health (SRH) [5].

This evolution from "health-based" to "rights-based" approaches represents a fundamental change in philosophy from a deficit-based model (primarily preventing problems) to an asset-based model that empowers individuals. CSE is not merely about disseminating information, but about nurturing critical thinking, fostering autonomy, and developing personal agency. These capacities enable young people to navigate complex social and cultural contexts effectively. It extends beyond individual health outcomes, influencing broader societal aspects such as school social climates and a country’s socioeconomic development. Effective implementation of CSE can make a more equitable and compassionate society by empowering individuals and minor communities [3].

The multifaceted landscape of modern sexuality education

Pedagogical Approaches and Age-Appropriate Strategies

Age-appropriateness, medical accuracy, and cultural relevance are important for effective CSE. It is delivered incrementally, establishing foundational knowledge and skills from an early age, with new information building upon prior learning at advanced levels [1].

Age-specific strategies are crucial for tailoring content to developmental stages. For babies and toddlers, education focuses on naming private body parts, allowing for natural exploration, highlighting bodily differences, explaining bodily functions, and introducing family rules regarding nudity. For children aged 3-5 years, the approach includes identifying body parts and functions, differentiating private from public, respecting privacy as well as body autonomy, emphasizing the importance of consent, and introducing basics of reproduction. As children progress to 6-8 years, the curriculum introduces internal reproductive organs, discusses puberty and associated emotional changes, explores similarities and differences between male and female bodies, teaches refusal skills and body safety, and normalizes adult sexual activity as a natural and healthy part of life. For 9-12-year-olds, more detailed information is provided on puberty, first periods and ejaculations, fertility, sexual intercourse, and basic STI and pregnancy prevention, alongside discussions about personal values and beliefs regarding sexuality. In high school (13-16-year-olds), topics expand to include contraception, safer sex practices, sexual decision-making, communication skills, various relationship types, strategies for coping with peer pressure, and the influence of legal, cultural, religious, and media factors on sexual health [6].

Effective pedagogical methods for CSE are interactive, participatory, and focused on skill-building. Role play allows students to practice scenarios, develop decision-making skills, enhance self-confidence, and gain understanding of diverse perspectives within a safe environment. Small-group activities help in collaborative learning and deeper comprehension. Class discussions are important in introducing factual information, correcting misinformation, promoting the use of proper terminology, and cultivating respectful dialogue [1]. Peer education programs are also effective as young people value and retain sexual health information more readily when delivered by their peers [7]. A core focus of these programs is skill-building, encompassing personal competencies such as communication, boundary setting, negotiation, and decision-making [1]. These skills are integrated across all grade levels, progressing from basic communication of personal boundaries in early childhood to complex discussions about consent and STI/HIV status in adolescence. This comprehensive approach ensures that young people are well-informed and empowered to make healthy choices and navigate complex social situations, leading to improved long-term physical and mental health outcomes.

Fostering Emotional Well-Being, Healthy Relationships, and Consent

CSE takes care of emotional well-being by equipping young people with strategies to understand and manage strong emotions, cope with feelings, and cultivate self-esteem. It focuses on the development of healthy relationships through discussions on the evolution of friendships, identifying the characteristics of a good friend, recognizing and addressing bullying, and repairing relational conflicts. CSE also emphasizes respectful social and sexual relationships by including discussions on diverse family structures and same-sex relationships, thereby normalizing and validating a wide spectrum of human connections [1].

Consent education is an indispensable component, teaching individuals that they have the right to make their own decisions regarding sexual activity. This education underscores the importance of consent being ongoing, enthusiastic, and clearly communicated. CSE also plays a vital role in preventing child sexual abuse or intimate partner violence by teaching essential skills involving body safety, refusal techniques, and how to identify and avoid coercive situations. The integration of consent education from early childhood demonstrates consent as a fundamental principle of bodily autonomy and respect applicable to all social interactions, not only sexual ones [8]. This helps establish a lifelong foundation for healthy relationships and reduces vulnerability to various forms of abuse. By teaching consent as a universal life skill, CSE contributes to cultivating a broader societal culture of respect, which in turn helps to reduce instances of violence and promotes healthier interpersonal dynamics across all contexts.

Integrating Digital Tools and Technology in Sex Education

As adolescents increasingly access sexual information, including pornography, through online platforms, CSE must adapt to this digital environment by addressing critical aspects of cyber safety and promoting responsible mobile phone use. Media literacy education is an important approach in CSE. It equips young people with critical thinking skills to evaluate the persuasive and often unhealthy messages about sex and relationships prevalent in digital media, including pornography. This critical engagement can help to mitigate the acceptance of traditional gender roles, the objectification of individuals, and the normalization of dating violence, which are frequently perpetuated by unfiltered online content [9].

Digital tools and online resources are also being developed for educational purposes, including web-based applications, educational websites, mobile applications, and YouTube channels. Online sexuality education interventions reach populations that have been left out by traditional educational methods [9]. Media literacy transforms passive consumers of online content into critical evaluators, essential for protecting youth in the digital world, where information (both accurate and inaccurate) is readily accessible.

A pilot study on an online intervention for high school students revealed that the program had a good initial effect on sexual health behaviors. Participants reported a decrease in self-reported dating violence, an increase in condom use, and an increase in sexual awareness [10].

According to a randomized controlled trial in the UK, peer discussion and debate increased as a result of a digital game created to promote relationships and sex education. The ability of digital technologies to develop critical abilities is demonstrated by the notable improvement in learner confidence in recognizing and preventing sexual coercion among those who engaged with the game [11].

Cultural, societal, and policy dynamics

Global Perspectives and Comparative Models

Globally, there is a significant policy commitment to sexuality education, with 85% of 155 surveyed countries reporting policies or laws related to CSE [12,13]. CSE is gaining worldwide acceptance and political commitment, as evident by notable progress in its development and integration into formal school settings across regions such as Europe and Central Asia [1]. International organizations, including the United Nations (UN), United Nations Educational, Scientific and Cultural Organization (UNESCO), United Nations Population Fund (UNFPA), and World Health Organization (WHO), advocate for CSE as the most effective and preferred method for enhancing young people's SRH and rights [14,15]. Frameworks such as the "Standards for Sexuality Education in Europe" provide comprehensive guidance for developing holistic sexuality education curricula [5]. A state's failure to ensure publicly available, accessible, and accurate SRH information within educational curricula is viewed as a violation of its obligations under the human right to health, as supported by international agreements like the 1994 International Conference on Population and Development (ICPD) Programme of Action, with the UN Committee on Economic, Social and Cultural Rights [16].

There are still significant implementation challenges due to the disparity between policy intentions and practical execution. This indicates that merely enacting a policy is insufficient; effective implementation necessitates dedicated resources, robust coordination among stakeholders, and the proactive overcoming of various systemic barriers. This warrants robust national strategies, clear mandates, and sustained funding to translate policy objectives into beneficial educational outcomes for young people worldwide [17].

Navigating Controversies and Implementation Challenges

Sexuality education is embedded within specific cultural contexts, leading to diverse perceptions and varied approaches globally. Traditional values, religious beliefs, and political authorities significantly influence sex education, often resulting in conflicts with modern educational concepts [18]. In multicultural societies, inconsistencies in beliefs about sex education are common, where traditional viewpoints may advocate for abstinence-only approaches and perceive modern CSE as potentially corrupting youth [19]. Family dynamics, technological advancements, racial and ethnic backgrounds, peer influences, and religious or spiritual affiliations are the factors affecting the implementation of CSE [18].

Controversies and gaps persist within the field. The historical origins of the "sexual rights revolution" and the CSE movement have been controversially linked to alleged fraudulent research, highlighting profound ideological divisions [4]. The integration of modern concepts, such as contraception, with traditional or religious moral frameworks (e.g., abstinence-only education) often creates moral conflict and contradictory messages, thereby limiting the effectiveness of CSE [18]. In countries like the United States, inconsistent standards prevail, with sex education topics and practices varying significantly by state and school district [20]. Only 39 states mandate sex or HIV education, and a substantial number of adolescents do not receive instruction that meets minimum standards [14,21]. Furthermore, the timing of education is often problematic, as it frequently occurs too late for youth who initiate sexual activity at younger ages [21].

Significant gaps exist for gender and sexual minority youth (GSMY). School-based sex education often remains heteronormative, focusing on penile-vaginal intercourse, lacking relevant information on diverse sexual acts and gender identity [22]. This leads to poor mental health outcomes, increased bullying, uninformed sexual decisions, and higher-risk behaviors among GSMY [22,23]. Implementation challenges are more in low- and middle-income countries (LMICs). Insufficient and fragmented funding, lack of coordination among government bodies and non-governmental organizations (NGOs), and the absence of monitoring and evaluation systems affect effective implementation. Additionally, centralized curricula often lack sufficient adaptation to diverse local contexts, and teachers frequently lack the necessary training to effectively tailor the content [17].

Overcoming these controversies requires more than just presenting scientific evidence; it demands robust community engagement, transparent communication, and culturally sensitive adaptation that acknowledges and addresses underlying societal anxieties and moral frameworks. The challenges observed in LMICs point to systemic rather than isolated issues, reflecting deeper problems in governance and resource allocation. Therefore, effective CSE implementation, particularly in diverse and resource-constrained settings, necessitates a multisectoral approach involving governments, civil society, and communities, with clear mandates, sustained funding, and robust monitoring systems [17].

Impact on public health and equity

Preventing STIs/HIV and Improving Reproductive Health Outcomes

CSE plays a crucial role in promoting responsible reproductive health behaviors, preventing unintended pregnancies, and reducing the incidence of STIs [3,24]. Research indicates that CSE programs can lead to a substantial reduction in unintended pregnancies by 25%-30% and a decrease in STI rates by 40% [3]. Effective CSE enhances young people's knowledge and improves their attitudes regarding SRH behaviors. This leads to positive outcomes such as delaying the age of sexual debut, increasing the use of condoms and other contraceptives when sexually active, and decreasing engagement in risk-taking behaviors [1].

CSE, when incorporated with gender and power dynamics within relationships’ discussions, is more likely to prevent unintended pregnancies and STIs. This suggests that merely providing biological facts or even practical skills is insufficient if underlying societal norms and power imbalances are not addressed. This implies that CSE should be transformative, actively challenging harmful gender norms and empowering individuals to negotiate equitable relationships [3]. By doing so, it maximizes its public health impact, extending far beyond simple risk reduction to foster genuine well-being and equality.

LGBTQ+ Inclusive Sexuality Education

Research consistently demonstrates significant health disparities experienced by lesbian, gay, bisexual, transgender, and queer/questioning (LGBTQ+) youth. These disparities include higher risks for suicidal thoughts, substance use, forced sexual encounters, dating violence, and STIs, when compared to their heterosexual peers [22,23,25]. The absence of LGBTQ+ inclusive sex education leads to the lack of critical information and reliance on unreliable sources like the internet or pornography [22].

Best practices for LGBTQ+ inclusive sexuality education emphasize the use of inclusive language, such as gender-neutral terms, and the consistent integration of LGBTQ+ examples and representation throughout the curriculum [22,26]. A key pedagogical approach involves teaching about body parts and their functions rather than making assumptions about identities. Inclusive education actively promotes safer school environments by reducing bullying, discrimination, and harassment, and fosters greater empathy among all students [22].

Sexuality Education for Children and Adolescents With Disabilities

Individuals with intellectual and developmental disabilities (I/DD) possess unique sexual needs and concerns that can be effectively addressed through tailored CSE [3,27]. Such education has been shown to reduce inappropriate sexual behaviors, enhance decision-making skills in situations involving sexual abuse, and improve overall social competencies [3]. Holistic sexual health education for I/DD youth should be designed to help them understand their identity and rights, how to express themselves, and how to cultivate healthy relationships [27]. The implementation of specialized teaching tools and adaptive pedagogical approaches is essential for this population [3,28]. Curricula developed for I/DD must be rights-based, trauma-informed, and explicitly inclusive and affirming of all genders, sexual orientations, races, and cultures [27].

The significant health disparities faced by LGBTQ+ youth and individuals with disabilities are directly linked to the historical exclusion and irrelevance of traditional sexuality education for these groups. The emphasis on using "inclusive language," providing "representation," and "teaching about parts, not assumed identities" for LGBTQ+ youth, alongside "adaptive approaches" and "rights-based, trauma-informed" curricula for individuals with I/DD, demonstrates that inclusivity is not merely an ethical consideration but a pragmatic necessity for achieving health equity. When education is not specifically tailored to the diverse experiences of all young people, it can cause harm. A truly CSE must, therefore, actively dismantle biases, ensuring that all young people receive relevant, affirming, and accurate information.

Teacher preparedness and training

The preparedness of educators is critical for the effective delivery of CSE [27]. Significant challenges in this area include a lack of specialized training for educators, insufficient policy support, gaps in teachers' foundational knowledge, and a general sense of embarrassment or discomfort when discussing sex-related topics [29,30]. Consequently, the delivery of CSE can become ambiguous, hindering student comprehension and diminishing overall effectiveness [29].

To address these challenges, various professional development opportunities are being implemented. Virtual professional development (Virtual PD) programs utilize simulated classroom environments where teachers can practice instructing student avatars and receive real-time coaching. These trainings often focus on practical skills, such as creating inclusive classrooms for transgender and gender-expansive students and employing inclusive language [29]. Asynchronous, instructor-led online learning experiences are also available, designed to enhance educators' capacity to teach about healthy relationships, consent, technology, and social media [30]. Training programs also address common concerns from parents and include specific modules for LGBTQ+ inclusivity and awareness of unconscious biases related to race, ethnicity, and culture [29,31]. Sufficient theoretical and practical training is vital for motivating teachers and significantly increasing their self-confidence in delivering CSE effectively [29,32]. Investing in teacher preparedness is, therefore, not merely an administrative consideration but a strategic imperative for improving public health outcomes through CSE.

## Conclusions

CSE is a critical public health intervention that has evolved from a narrow focus on risk prevention to a holistic, rights-based approach encompassing cognitive, emotional, physical, and social aspects of sexuality. Evidence consistently demonstrates its efficacy in reducing unintended pregnancies and STIs, fostering healthy relationships, and promoting consent.

However, the field faces significant challenges, including deep-seated cultural and religious controversies, inconsistent policy implementation, and critical gaps in addressing the unique needs of marginalized populations like LGBTQ+ youth and individuals with disabilities. The success of CSE is based on a multipronged strategy: sustained political commitment, dedicated funding and robust monitoring, culturally sensitive adaptations that engage diverse communities, and comprehensive teacher training programs that build both knowledge and confidence. Furthermore, integrating media literacy into curricula is paramount in this digital world. By embracing inclusivity and addressing systemic barriers, CSE can fully realize its potential as a transformative force for individual well-being and broader public health.
